# Combination oral antiangiogenic therapy with thalidomide and sulindac inhibits tumour growth in rabbits

**DOI:** 10.1038/sj.bjc.6690020

**Published:** 1999-09-24

**Authors:** H M W Verheul, D Panigrahy, J Yuan, R J D'Amato

**Affiliations:** Department of Surgery, Children's Hospital, Harvard Medical School, 300 Longwood Avenue, Boston, MA 02115, USA; Department of Ophthalmology, Harvard Medical School, 300 Longwood Avenue, Boston, MA 02115, USA

**Keywords:** basic fibroblast growth factor, vascular endothelial growth factor, corneal neovascularization, non-steroidal anti-inflammatory drug, cyclo-oxygenase

## Abstract

Neovascularization facilitates tumour growth and metastasis formation. In our laboratory, we attempt to identify clinically available oral efficacious drugs for antiangiogenic activity. Here, we report which non-steroidal anti-inflammatory drugs (NSAIDs) can inhibit corneal neovascularization, induced by basic fibroblast growth factor (bFGF) or vascular endothelial growth factor (VEGF). This antiangiogenic activity may contribute to the known effects of NSAIDs on gastric ulcers, polyps and tumours. We found that sulindac was one of the most potent antiangiogenic NSAIDs, inhibiting bFGF-induced neovascularization by 50% and VEGF-induced neovascularization by 55%. Previously, we reported that thalidomide inhibited growth factor-induced corneal neovascularization. When we combined sulindac with thalidomide, we found a significantly increased inhibition of bFGF- or VEGF-induced corneal neovascularization (by 63% or 74% respectively) compared with either agent alone (*P*< 0.01). Because of this strong antiangiogenic effect, we tested the oral combination of thalidomide and sulindac for its ability to inhibit the growth of V2 carcinoma in rabbits. Oral treatment of thalidomide or sulindac alone inhibited tumour growth by 55% and 35% respectively. When given together, the growth of the V2 carcinoma was inhibited by 75%. Our results indicated that oral antiangiogenic combination therapy with thalidomide and sulindac may be a useful non-toxic treatment for cancer. © 1999 Cancer Research Campaign


*British Journal of Cancer* (1999) **79**(1), 114–118
© 1999 Cancer Research Campaign

### Combination oral antiangiogenic therapy with thalidomide and sulindac inhibits tumour growth in rabbits

**HMW Verheul[1], D Panigrahy[1], J Yuan[1] and RJ D'Amato[1,2]**

[1]Department of Surgery, Children's Hospital and [2]Department of Ophthalmology, Harvard Medical School, 300 Longwood Avenue, Boston MA 02115, USA

**Summary** Neovascularization facilitates tumour growth and metastasis formation. In our laboratory, we attempt to identify clinically available oral efficacious drugs for antiangiogenic activity. Here, we report which non-steroidal anti-inflammatory drugs (NSAIDs) can inhibit corneal neovascularization, induced by basic fibroblast growth factor (bFGF) or vascular endothelial growth factor (VEGF). This antiangiogenic activity may contribute to the known effects of NSAIDs on gastric ulcers, polyps and tumours. We found that sulindac was one of the most potent antiangiogenic NSAIDs, inhibiting bFGF-induced neovascularization by 50% and VEGF-induced neovascularization by 55%. Previously, we reported that thalidomide inhibited growth factor-induced corneal neovascularization. When we combined sulindac with thalidomide, we found a significantly increased inhibition of bFGF- or VEGF-induced corneal neovascularization (by 63% or 74% respectively) compared with either agent alone ($P < 0.01$). Because of this strong antiangiogenic effect, we tested the oral combination of thalidomide and sulindac for its ability to inhibit the growth of V2 carcinoma in rabbits. Oral treatment of thalidomide or sulindac alone inhibited tumour growth by 55% and 35% respectively. When given together, the growth of the V2 carcinoma was inhibited by 75%. Our results indicated that oral antiangiogenic combination therapy with thalidomide and sulindac may be a useful non-toxic treatment for cancer.

**Keywords:** basic fibroblast growth factor; vascular endothelial growth factor; corneal neovascularization; non-steroidal anti-inflammatory drug; cyclo-oxygenase

Angiogenesis is essential for tumours to grow beyond 2–3 $mm^3$ in size (Folkman, 1975, 1989). It has been demonstrated that specific angiogenesis inhibitors can inhibit tumour growth in animal models (Folkman, 1997). A continuing goal of our laboratory is to develop therapies for cancer patients that specifically block the angiogenic process.

Although angiogenesis is important for tumour growth and fetal development, it is also involved in inflammation and wound healing (Folkman, 1995). Anti-inflammatory agents are known to interfere with wound healing and pathological angiogenic disorders like rheumatoid arthritis and osteoarthritis (Insel, 1996). Anti-inflammatory agents, such as dexamethasone and NSAIDs, inhibit inflammatory-induced angiogenesis, caused by cauterization or by laser (Haynes et al, 1989; Sakamoto et al, 1995). Except for steroidal agents and indomethacin, anti-inflammatory agents have not been shown to inhibit non-inflammatory angiogenesis (Folkman and Ingber, 1987; Silverman et al, 1988).

In animal tumour models, NSAIDs, such as indomethacin, have been reported to inhibit tumour growth and to potentiate the effect of chemotherapy or radiotherapy (Fulton, 1984; Lynch et al, 1978; Teicher et al, 1994). Clinically, it has been observed that intake of NSAIDs correlates with a reduced degree of neovascularization in the granulation tissue of patients with gastroduodenal ulcers (Hudson et al, 1995). Sulindac also caused regression in the number and size of adenomas in patients with familial adenomatous polyposis (Labayle et al, 1991; Giardiello et al, 1993).

In this study, we tested clinically available, oral anti-inflammatory drugs for their antiangiogenic activity in the corneal neovascularization assay, a growth factor-induced non-inflammatory angiogenesis model (Kenyon et al, 1996). We have previously demonstrated that thalidomide can inhibit growth factor-induced angiogenesis in rabbits and mice (D'Amato et al, 1994; Kenyon et al, 1997). Therefore, we investigated whether NSAIDs were able to enhance the antiangiogenic effect of thalidomide. Based on the results of the corneal neovascularization assay and as sulindac is a safe drug for long-term treatment (Insel, 1996), we also determined the anti-tumour effect of the oral combination of thalidomide and sulindac in a rabbit tumour model.

### MATERIALS AND METHODS

### Drugs

The NSAIDs aspirin, phenidone, quercetin, esculetin, nordihydroguiaretic acid (NDGA), acetominophen, ibuprofen, sulindac and indomethacin were purchased from. Sigma Chemical Co. (St Louis, MO, USA). The following compounds – sulindac sulphone and sulindac sulphide (metabolites of sulindac), a specific cyclooxygenase 2 inhibitor (NS398), methylheptyl imidazole and furegrelate sodium (thromboxane inhibitors) and SKF525AHCL, an inhibitor of thromboxane while inducing prostaglandin production by endothelial cells – were all obtained from Biomol Research Laboratories (Plymouth Meeting, PA, USA). In mice, drugs were given at the highest tolerated dose, determined as the maximum dose not associated with signs of toxicity, as measured by weight

*Received 2 March 1998*
*Revised 12 May 1998*
*Accepted 14 May 1998*

*Correspondence to:* R D'Amato, Department of Surgery, Children's Hospital, Harvard Medical School, 300 Longwood Avenue, Boston MA 02115, USA

**Table 1** Inhibitory effect of non-steroidal anti-inflammatory drugs in the corneal neovascularization assay stimulated by bFGF or VEGF

| NSAID | Dose (mg $kg^{-1}$) | Per cent inhibition | | *n* | *P*-value |
|---|---|---|---|---|---|
| | | bFGF | VEGF | | |
| Acetaminophen | 100[a] | 0 | – | 8 | ns |
| Aspirin | 10–160[a] | 0–11 | – | 8 | ns |
| NDGA | 25[b] | 30 | – | 8 | ns |
| Esculetin | 200[c] | 15 | – | 8 | 0.02 |
| Phenidone | 100[b] | 17 | – | 8 | < 0.01 |
| Quercetin | 300[c] | 18 | – | 8 | < 0.01 |
| Ibuprofen | 25[b] | 6 | 8 | 23/8 | ns/ns |
| Ketoprofen | 80[b] | 30 | 41 | 8/8 | < 0.01 |
| Indomethacin | 5[b] | 59 | 61 | 15/21 | < 0.01/< 0.01 |
| Sulindac | 25[b] | 50 | 55 | 15/15 | < 0.01/< 0.01 |

[a]oral; [b]s.c.; [c]i.p. Inhibitory effect is expressed in percentage representing the area of corneal neovascularization induced by either bFGF or VEGF compared with controls (*n* = 8 per experiment) of the experiments in which that particular drug was tested; *n* = the number of eyes that were tested. Drugs were given once daily s.c., i.p. or orally.

loss, hair loss, infection and lethargy during 5 or 6 days of treatment. We screened these drugs for antiangiogenic activity by either s.c. or i.p. injections because we wanted to avoid any variation in the assay due to differences in absorption. After this initial screen, we confirmed the inhibitory effect of the most potent agents when orally administered.

### Mice and rabbits

Six- to eight-week-old C57B16 male mice were obtained from Jackson Laboratories (Bar Harbor, MA, USA). New Zealand White female rabbits (1.5 kg) were ordered from Charles River (Wilmington, MA, USA). Both species were housed in the animal research facilities of Children's Hospital.

### Corneal micropocket assay

In the stroma of the mouse cornea adjacent to the limbus, pellets were implanted with bFGF or VEGF as described previously (Haynes et al, 1989). In brief, after anaesthetizing the mice, $0.4 \times 0.4$ $mm^2$ pockets were made in the cornea. Subsequently, 80-ng bFGF or 160-ng VEGF pellets were implanted 1.0–1.2 mm or 0.5–0.7 mm from the limbal vessels respectively. Then, erythromycin was topically applied (E Fougera, Melville, NY, USA). The vascular response to the bFGF or VEGF pellets was measured 5 or 6 days after implantation, respectively, by maximal vessel length and number of clock hours of neovascularization. The area of corneal neovascularization was calculated by using a modified formula of a half ellipse, that best approximates the area of neovascularization: area ($mm^2$) = [ $\pi \times$ clock hours $\times$ length (mm) $\times$ 0.2 mm].

### Tumour assay

Female New Zealand White rabbits were used for propagating the V2 carcinoma. This tumour originates from a Shope virus-induced papilloma (Kidd and Rous, 1940). Small $0.5 \times 0.5$ $cm^2$ pieces were implanted intramuscularly in the right thigh. Treatment was started at day 10 after tumour implantation, when the mean volume of the tumour was 6 $cm^3$. Rabbits were sacrificed 17 days after the start of treatment when the mean volume of control tumours was 120 $cm^3$. Length and width of tumours were measured to calculate tumour volume with the formula: length $\times$ $(\text{width})^2$ $\times$ 0.52 = tumour volume. All experiments were conducted in accordance with the Animal Care and Use Committee.

### Immunohistochemistry

Tumour tissues were fixed in Carnoy's fixative overnight and embedded in paraffin according to standard histological procedures. Carnoy's fixed tissue sections (5–8 µm) were pretreated with 2 µg $ml^{-1}$ proteinase K (Boehringer Mannheim, Mannheim, Germany) at 37°C for 15 min before staining with a goat polyclonal antibody against human von Willebrand factor (1:1500 dilution; Incstar, Stillwater, MN, USA). Positive staining was detected by incubating sequentially with a biotinylated horse anti-goat secondary antibody (Vector Laboratories, Burlingame, CA, USA), avidin–horseradish peroxidase, chromagen and counterstained with haematoxylin. Microvessel density was determined by light microscopy according to the procedure of Weidner et al (1991). Each count was expressed as the number of microvessels identified within a selected 250× field. At least three separate 250× fields were analysed for each tumour specimen.

### Statistical analysis

The unpaired Student's *t*-test was used to test whether inhibitory activity was significantly different from controls. ANOVA (Instat Mac package) was performed to test whether combination treatment was significantly different from either agent alone. Significant difference was determined as *P*-value < 0.05.

### RESULTS

### Mouse corneal micropocket assay

Inhibition of corneal neovascularization in mice by single anti-inflammatory drugs ranged from 0% to 60% in both bFGF and VEGF assays, summarized in Table 1. Sulindac (25 mg $kg^{-1}$ $day^{-1}$ s.c.) and indomethacin (5 mg $kg^{-1}$ $day^{-1}$ s.c.) were the most potent inhibitors of angiogenesis induced by bFGF [50% ($n = 15$, $P < 0.01$) and 59% ($n = 21$, $P < 0.01$) respectively] and by VEGF

© Cancer Research Campaign 1999

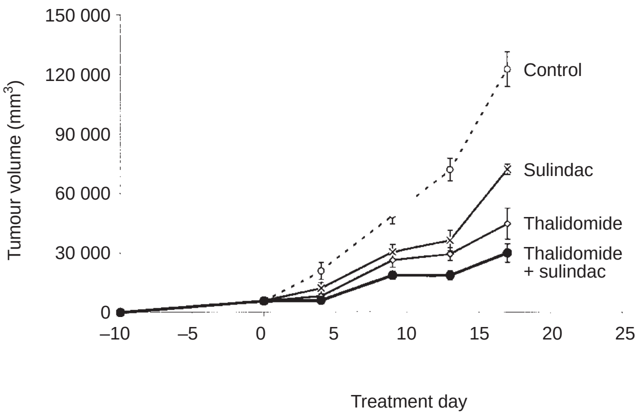

**Figure 1** Effect of orally administered thalidomide and sulindac on tumour growth of V2 carcinoma in rabbits. Ten days after implantation of a tumour piece in the right thigh of New Zealand White female rabbits, treatment with methylcellulose (*n* = 13), sulindac (*n* = 5), thalidomide (*n* = 14) or the combination of thalidomide and sulindac (*n* = 10) was started for 17 days. The combination of thalidomide and sulindac inhibited tumour growth by 75% and was significantly different ($P < 0.05$) from either agent alone or the control group. Oral treatment with sulindac or thalidomide inhibited tumour growth by, respectively, 35% ($n = 5$, $P < 0.01$) and 55% ($n = 14$, $P < 0.01$). The data were collected in three separate experiments and this figure shows a representative experiment (thalidomide, 200 mg $kg^{-1}$ p.o.; sulindac, 60 mg $kg^{-1}$ $day^{-1}$ p.o.; $n = 5$ per group. Each bar represents the standard error of the mean

[55% ($n = 15$, $P < 0.01$) and 61% ($n = 21$, $P < 0.01$) respectively]. Orally administered sulindac (30 mg $kg^{-1}$ twice daily) and indomethacin (5 mg $kg^{-1}$ $day^{-1}$) caused a similar inhibition of bFGF-induced neovascularization as the subcutaneous dosing regime ($n = 8$, $P < 0.01$). Ketoprofen and ibuprofen were less inhibitory for either bFGF- or VEGF-induced neovascularization. In addition, the lipoxygenase inhibitors esculetin, quercetin, phenidone and NDGA were less inhibitory in the bFGF-induced neovascularization corneal pocket assay. Aspirin and acetaminophen had no inhibitory effect.

When sulindac (25 mg $kg^{-1}$ $day^{-1}$) was administered in combination with thalidomide (200 mg $kg^{-1}$ $day^{-1}$), the combined inhibitory effect was significantly enhanced to 63% ($n = 15$, $P < 0.01$) for bFGF and 74% ($n = 16$, $P < 0.01$) for VEGF (see Table 2). Indomethacin, which has similar antiangiogenic effects as sulindac as a single agent, did not enhance the inhibitory effect of thalidomide [67% ($n = 15$, $P > 0.05$) for bFGF and 61% ($n = 21$, $P > 0.05$) for VEGF].

Sulindac is metabolized in vivo to the active metabolite sulindac sulphide and the inactive metabolite sulindac sulphone (with regard to the inhibitory activity of prostaglandin synthesis) (Duggan et al, 1977). Sulindac sulphide inhibited bFGF-induced neovascularization to a maximum of 34% (25 and 50 mg $kg^{-1}$ $day^{-1}$ s.c., $n = 8$ for each experiment, $P < 0.01$), whereas sulindac sulphone inhibited 31% (25 mg $kg^{-1}$ $day^{-1}$ s.c., $n = 16$, $P < 0.01$).

The specific thromboxane inhibitors, carbomethylheptyl imidazole and furegrelate sodium, inhibited bFGF-induced neovascularization by 32% ($n = 6$, $P < 0.01$) and 22% ($n = 16$, $P < 0.01$), respectively, at the highest tolerated dose (40 mg $kg^{-1}$ $day^{-1}$ s.c.). In addition, SKF525AHCL, an agent that inhibits thromboxane production but induces prostaglandin production, showed 25% inhibition ($n = 8$, $P < 0.01$). The specific cyclooxygenase 2 inhibitor NS-398 inhibited 25% at the highest tolerated dose of 20 mg $kg^{-1}$ $day^{-1}$ s.c. ($n = 16$, $P < 0.01$).

**Table 2** Inhibitory effect of sulindac if combined with thalidomide in the corneal neovascularization assay stimulated by bFGF or VEGF

| Drug | Dose (mg $kg^{-1}$) | Per cent inhibition | | *n* |
|---|---|---|---|---|
| | | bFGF | VEGF | |
| Thalidomide | 200 | 41 | 40 | 31/39 |
| Sulindac | 25 | 50 | 55 | 15/15 |
| Thalidomide + sulindac | 200 + 25 | 63[a] | 74[a] | 15/16 |

[a]Inhibitory effect is significantly different from either agent alone ($P < 0.01$, tested by ANOVA). Inhibitory effect is expressed in percentage representing the area of corneal neovascularization induced by either bFGF or VEGF compared with controls ($n = 8$ per experiment) of the two experiments in which the drugs were tested; $n$ = the number of eyes that were tested with bFGF/VEGF. Sulindac was given once daily s.c. and thalidomide once daily i.p.

### Rabbit V2 carcinoma model

V2 carcinoma, implanted in the thighs of control rabbits, grew in 27 days to about 70 g, which equalled 120 $cm^3$ in volume, as shown in Figure 1. Oral treatment with sulindac (60 mg $kg^{-1}$ $day^{-1}$) or thalidomide (200 mg $kg^{-1}$ $day^{-1}$) inhibited tumour growth by, respectively, 35% ($n = 5$, $P < 0.01$) and 55% ($n = 14$, $P < 0.01$), as shown in Figure 1. When the two drugs were combined, inhibition of tumour growth was enhanced to 75% ($n = 10$, $P < 0.05$, see Figure 1). A lower dose of 25 mg $kg^{-1}$ $day^{-1}$ of orally administered sulindac failed to inhibit tumour growth and did not enhance the inhibitory effect of thalidomide (data not shown). Weight loss, lethargy or hair loss was not observed during treatment.

Microvessel density is a parameter for tumour-induced angiogenesis (Folkman, 1995). To determine whether this therapy indeed affects tumour-induced neovascularization, we performed immunohistochemical staining on these tumour tissues with a polyclonal goat antibody against Von Willebrand factor that stains the endothelium of rabbits (Tanaka et al, 1994). A significantly reduced microvessel density (m.v.d.) was observed in the oral combination therapy (control m.v.d. = 32 ± 4 vs sulindac/thalidomide therapy m.v.d. = 14 ± 4; $n = 5$, $P < 0.01$, per 200× high-power field).

### DISCUSSION

The role prostaglandins play in neovascularization and tumour growth is not clear, but anti-tumour activity of prostaglandin synthetase inhibitors has been previously reported (Fulton, 1984; Lynch et al, 1984; Teicher et al, 1994). The potential role of the arachidonic acid cascade for tumour growth and metastases formation has been reviewed previously by Marnett (1992). Proposed explanations for the inhibitory effects of prostaglandin synthetase inhibitors on tumour growth included an antimutagenic effect, a direct inhibitory effect on tumour cell proliferation and prostaglandin production or an immunomodulating effect. Alternatively, we hypothesized that prostaglandin synthetase inhibitors may act by inhibiting angiogenesis and found that sulindac and indomethacin were effective inhibitors of growth factor-induced neovascularization.

Because sulindac and indomethacin are relatively selective for prostaglandin H synthetase 1 (COX-1) compared with the others we tested (Meade et al, 1993; Mitchell et al, 1993; Smith et al, 1994), our results suggests that COX-1 is important for non-inflammatory neovascularization. However, although sulindac and

© Cancer Research Campaign 1999

indomethacin are relatively selective for COX-1, they are still potent inhibitors of prostaglandin H synthetase 2 (COX-2). NS398, a specific COX-2 inhibitor, also inhibited corneal neovascularization, further indicating that both COX-1 and COX-2 are involved. Aspirin, an irreversible cyclooxygenase inhibitor, lacked an antiangiogenic effect. This absence of activity may be related to the observation that the irreversible inhibition of cyclooxygenase synthetases by aspirin resulted in a compensatory production of COX-2 with bioactivity (Karim et al, 1995). Still, we cannot rule out that some differences in antiangiogenic activity of the NSAIDs were caused by a difference in pharmacokinetics.

Inhibitors of thromboxane (an enzyme that is exclusively found in platelets) inhibited bFGF-induced corneal neovascularization. This finding suggests that platelets may play a role in angiogenesis, presumably because of their growth factor release upon activation. Indeed, it has been reported that thrombopenia inhibits tumour angiogenesis (Peterson, 1996).

The action of sulindac and its metabolites on corneal neovascularization suggests that regression of tumours in patients with familial adenomatous polyposis (FAP) (Labayle et al, 1991; Giardello et al, 1993) may be in part mediated through blocked angiogenesis. Indeed, significantly higher vessel counts have been found in adenomas compared with normal mucosa of the colon, whereas colon carcinomas showed higher vessel counts than adenomas. This suggests that angiogenesis may be important for polyp formation and for the transition to neoplasia (Bossi et al, 1995). We assumed that the inhibitory effect of sulindac was mainly due to an inhibition of cyclooxygenases. However, the inactive metabolite of sulindac, sulindac sulphone, which does not inhibit cyclooxygenase and cannot be reformed to sulindac or sulindac sulphide (Duggan et al, 1977) also inhibited corneal neovascularization. It was in fact as potent as sulindac sulphide, the active metabolite. Sulindac sulphone and sulphide have both been shown to induce apoptosis in colon tumour cell lines independent of cyclooxygenase synthetase inhibitory activity (Hanif et al, 1996; Piazza et al, 1997). Furthermore, sulindac has recently been demonstrated in a mouse model of FAP to regress tumours independent of its effect on prostaglandin production (Chiu et al, 1997). Therefore, the antiangiogenic effects of sulindac in the corneal neovascularization assay seem to be, at least partially, mediated by other mechanisms not involving cyclooxygenases.

The search for non-toxic antiangiogenic therapies is one of the important aims of our research laboratory. Because of the considerable advantages of oral treatment above other treatments, we attempted to find clinically available oral drugs with potent antiangiogenic activity. It has been demonstrated that the growth of V2 carcinoma in rabbits can be inhibited by antiangiogenic agents (Gross et al, 1981; Kamei et al, 1993). In this study, we found 55% tumour growth inhibition by oral treatment of V2-carcinoma-bearing rabbits with thalidomide. Our finding is in contrast to two other recently published studies, in which thalidomide failed to inhibit primary tumour growth in mice (Gutman et al, 1996; Minchinton et al, 1996). We attributed this discrepancy to the well-known differences in metabolism of thalidomide in rodents compared with rabbits or humans (Schumacher et al, 1965, 1968). Teratogenic malformations were detected after oral administration of thalidomide to rabbits and humans, but not detected in mice (Plies, 1962; Szabo and Steelman, 1967). In mice, thalidomide is only antiangiogenic when given i.p. at a dose of 200 mg $kg^{-1}$ (Kenyon et al, 1997). Unfortunately, in these other negative rodent tumour studies, they used approximately 4 mg $kg^{-1}$ $day^{-1}$ i.p. (Minchinton et al, 1996) or 12–40 mg $kg^{-1}$ $day^{-1}$ p.o. of thalidomide (Gutman et al, 1996). However, despite the use of such a low dose (4 mg $kg^{-1}$ $day^{-1}$ i.p.) of thalidomide, there was still inhibition of growth of lung metastases in Lewis lung carcinoma-bearing mice (Minchinton et al, 1996).

When we combined thalidomide with sulindac in the corneal neovascularization assay, we found a significantly enhanced antiangiogenic effect of the combination compared with either agent alone. As thalidomide and sulindac can be given as long-term treatment, we tested the oral combination in the V2-carcinoma model and found that the combination can inhibit tumour vascularity by 56% and tumour growth by 75%. It is among the most effective tumour inhibitions observed by an oral antiangiogenesis therapy in our laboratory. In addition to the promising preliminary results of the phase 1 clinical trials of thalidomide in Kaposi sarcoma and glioblastoma patients (Fine et al, 1997; Little et al, 1997), we provide here a potential application for combination of oral antiangiogenic therapy.

### ACKNOWLEDGEMENTS

We thank E. Flynn for preparations and analysis of the histological sections. We thank T. Udagawa for discussions and review of the manuscript. This study was supported by a grant to the Children's Hospital from Entremed (Rockville, Maryland, USA). H.M.W.V. is a recipient of the Margot Mattheijssen-van der Voort Fellowship and of a grant of the Nijbakker Morra Society (The Netherlands).

### REFERENCES

Bossi P, Viale G, Lee AKC, Alfano RM, Coggi G and Bosari S (1995) Angiogenesis in colorectal tumors: microvessel quantitation in adenomas and carcinomas with clinicopathological correlations. *Cancer Res* **55**: 5049–5053

Chiu C, McEntee MF and Whelan J (1997) Sulindac causes rapid regression of preexisting tumors in *Min*/+ mice, independent of prostaglandin biosynthesis. *Cancer Res* **57**: 4267–4273

D'Amato RJ, Loughnan MS, Flynn E and Folkman J (1994) Thalidomide is an inhibitor of angiogenesis. *Proc Natl Acad Sci USA* **91**: 4082–4085

Duggan DE, Hooke KF, Risley EA, Shen TY and van Arman CG (1977) Identification of the biologically active form of sulindac. *J Pharm Exp Ther* **201**: 8–13

Fine HA (1997) A phase II trial of the antiangiogenic agent thalidomide in patients with recurrent high-grade gliomas (abstract). In *Thalidomide: Potential Benefits and Risks, Open Public Scientific Workshop*, p. 85. National Institute of Health: Bethesda, MD

Folkman J (1975) Tumor angiogenesis: a possible control point in tumor growth. *Ann Intern Med* **82**: 96–100

Folkman J (1989) What is the evidence that tumors are angiogenesis dependent? *J Natl Cancer Inst* **82**: 4–6

Folkman J (1995) Angiogenesis in cancer, vascular, rheumatoid and other disease. *Nature Med* **1**: 27–31

Folkman J (1997) Antiangiogenic therapy. In *Cancer, Principles and Practice of Oncology*. DeVita Jr VT, Hellman S and Rosenberg SA (eds), pp. 3075–3086. Lipincott Raven: New York

Folkman J and Ingber DE (1987) Angiostatic steroids: method of discovery and mechanism of action. *Ann Surg* **206**: 374–384

Fulton AM (1984) In vivo effects of indomethacin on the growth of murine mammary tumors. *Cancer Res* **44**: 2419–2420

Giardiello FM, Hamilton SR, Krush AJ, Piantodosi S, Hylind LM, Cleano P, Banker SV, Robinson CR and Offerhaus GJ (1993) Treatment of colonic rectal adenomas with sulindac in familial adenomatous polyposis. *N Engl J Med* **328**: 1313–1316

Gross J, Azizkhan RG, Biswas C, Bruns RR, Hsieh DST and Folkman J (1981) Inhibition of tumor growth, vasularization, and collagenolysis in the rabbit cornea by medroxyprogesterone. *Proc Natl Acad Sci USA* **78**: 1176–1180

© Cancer Research Campaign 1999 

Gutman M, Szold A, Ravid A, Lazouskas T, Merimsky O and Klausner JM (1996) Failure of thalidomide to inhibit tumor growth and angiogenesis in vivo. *Anticancer Res* **16**: 3673–3678

Hanif R, Pittas A, Feng Y, Koutsos MI, Qiao L, Staiano-Coico L, Shiff SI and Rigos B (1996) Effects of nonsteroidal anti-inflammatory drugs on proliferation and on induction of apoptosis in colon cancer cells by a prostaglandin-independent pathway. *Biochem Pharmacol* **52**: 237–245

Haynes WL, Proia AD and Klintworth GK (1989) Effect of inhibitors of arachidonic acid metabolism on corneal neovascularization in the rat. *Invest Ophthalmol Vis Sci* **30**: 1588–1593

Hudson N, Balsitis M, Everitt S and Hawkey CJ (1995) Angiogenesis in gastric ulcers: impaired in patients taking non-steroidal anti-inflammatory drugs. *Gut* **37**: 191–194

Insel PA (1996) Analgesic–antipyretic and antiinflammatory agents and drugs employed in the treatment of gout. In *The Pharmacological Basis of Therapeutics*. Hardman JG and Limbird LE (eds), pp. 617–658. The McGraw-Hill Companies: New York

Kamei S, Okada H, Inoue Y, Yoshioka T, Ogawa Y and Toguchi H (1993) Antitumor effects of angiogenesis inhibitor TNP-470 in rabbits bearing VX-2 carcinoma by arterial administration of microspheres and oil solution. *J Pharm Exp Ther* **264**: 469–474

Karim S, Habib A, Levy-Toledano S and Maclouf J (1995) Cyclooxygenases-1 and -2 of endothelial cells utilize exogenous or endogenous arachidonic acid for transcellular production of thromboxane. *J Biol Chem* **271**: 12042–12048

Kenyon BM, Voest EE, Chen C, Flynn E, Folkman J and D'Amato RJ (1996) A model of angiogenesis in the mouse cornea. *Invest Ophthalmol Vis Sci* **37**: 1625–1632

Kenyon BM, Browne F and D'Amato RJ (1997) Effects of thalidomide and related metabolites in a mouse corneal model of neovascularization. *Exp Eye Res* **64**: 971–978

Kidd JG and Rous P (1940) A transplantable rabbit carcinoma originating in a virus-induced papilloma and containing the virus in masked or altered form. *J Exp Med* **71**: 813–838

Labayle D, Fischer D, Vielh P, Drouhin F, Pariente A, Bories C, Duhamel A, Transset M and Attali P (1991) Sulindac causes regression of rectal polyps in familial adenomatous polyposis. *Gastroenterology* **101**: 635–639

Little R, Welles L, Wyvill K, Pluda J, Figg W, Tosato G and Yarchoan R (1997) Preliminary results of a phase II dose titration study of oral thalidomide in patients with HIV infection and Kaposi's sarcoma (abstract). In *Thalidomide: Potential Benefits and Risks, Open Public Scientific Workshop*, p. 91. National Institute of Health: Bethesda, MD

Lynch NR, Castes M, Astoin M and Salomon JC (1978) Mechanism of inhibition of tumour growth by aspirin and indomethacin. *Br J Cancer* **38**: 503–512

Marnett LJ (1992) Aspirin and the potential role of prostaglandins in colon cancer. *Cancer Res* **52**: 5575–5589

Meade EA, Smith WL and DeWitt DL (1993) Differential inhibition of prostaglandin endoperoxide synthetase (cyclooygenase) isozymes by aspirin and other non-steroidal anti-inflammatory drugs. *J Biol Chem* **268**: 6610–6614

Minchinton AI, Fryer KH, Wendt KR, Clow KA and Hayes MMM (1996) The effect of thalidomide on experimental tumors and metastases. *Anticancer Drugs* **7**: 339–343

Mitchell JA, Akarasereenont P, Thiemermann C, Flower RJ and Vane JR (1993) Selectivity of nonsteroidal antiinflammatory drugs as inhibitors of constitutive and inducible cyclooxygenase. *Proc Natl Acad Sci USA* **90**: 11693–11697

Peterson H (1996) Tumor angiogenesis inhibition by prostaglandin synthetase inhibitors. *Anticancer Res* **6**: 251–254

Piazza GA, Rahm AL, Krutzsch M, Sperl G, Paranka NS, Gross PH, Brendel K, Burt RW, Alberts OS and Paniukou R (1997) Antineoplastic drugs sulindac sulfide and sulfone inhibit cell growth by inducing apoptosis. *Cancer Res* **55**: 3110–3116

Pliess G (1962) Thalidomide and congenital abnormalities. *Lancet* **2**: 1128–1129

Sakamoto T, Soriano D, Nassaralla J, Murphy TL, Oganesian A, Spee C, Hinton DR and Ryan SJ (1995) Effect of intravitreal administration of indomethacin on experimental subretinal neovascularization in the subhuman primate. *Arch Ophthamol* **113**: 222–226

Schumacher H, Smith RL and Williams RT (1965) Metabolism of thalidomide: the fate of thalidomide and some of its hydrolysis products in various species. *Br J Pharmacol* **25**: 338–351

Schumacher H, Blake DA and Gilette JR (1968) Disposition of thalidomide in rabbits and rats. *J Pharm Exp Ther* **160**: 201–211

Silverman KJ, Lund DP, Zetter BR, Lainey LL, Shahood JA, Freiman DG, Folkman J and Burger AC (1988) Angiogenic activity of adipose tissue. *Biochem Biophys Res Commun* **153**: 347–352

Smith WL, Meade EA and DeWitt DL (1994) Interactions of PGH synthase isozymes-1 and -2 with NSAIDs. *Ann NY Acad Sci* **744**: 50–57

Szabo KT and Steelman RL (1967) Effects of maternal thalidomide treatment on pregnancy, fetal development, and mortality of the offspring in random-bred mice. *Am J Vet Res* **28**: 1823–1828

Tanaka H, Sukhova GK and Libby P (1994) Interaction of the allogeneic state and hypercholesterolemia in arterial lesion formation in experimental cardiac allografts. *Arteriosclerosis Thromb* **14**: 734–745

Teicher BA, Korbut TT, Menon K, Holden SA and Ara G (1994) Cyclooxygenase and lipoxygenase as modulators of cancer therapies. *Cancer Chem Pharm* **33**: 515–522

Weidner N, Semple JP, Welch WR and Folkman J (1991) Tumor angiogenesis and metastasis – correlation in invasive breast carcinoma. *N Engl J Med* **324**: 1–8

 *© Cancer Research Campaign 1999*
